# Supplementary material for: The Associations between Snack Intake and Cariogenic Oral Microorganism Colonization in Young Children of a Low Socioeconomic Status
Source: Nutrients. 2024 Apr 10;16(8):1113. doi: 10.3390/nu16081113 (PMC11054055; doi:10.3390/nu16081113)
Supplement: Supplementary file 1 [file nutrients-16-01113-s001.zip › nutrients-2951591-supplementary.pdf]

Supplementary Table S1. Associations between weighted snack intake and oral microorganism carriage from child aged 12 months to 24 months.

	Sweet Index_Weighted			Non-sweet Index_Weighted		
	OR	95% CI	p	OR	95% CI	p
S. mutans carriage__saliva	1.02	0.98, 1.05	0.29	1.00	0.99, 1.02	0.49
S. mutans carriage__plaque	1.01	0.98, 1.05	0.55	**1.02**	**1.00, 1.03**	**0.02**
Candida carriage__saliva	0.99	0.96, 1.02	0.44	1.01	1.00, 1.02	0.21
Candida carriage__plaque	0.99	0.96, 1.03	0.75	**1.01**	**1.00, 1.02**	**<0.05**

Supplementary Table S2. Interactions between weighted sweet/non-sweet indices and time of visit and their associations with oral microorganisms.

	S. mutans carriage__Saliva		*S. mutans* carriage__Plaque		*Candida* carriage__Saliva		*Candida* carriage__Plaque	
	OR (95%CI)	p	OR (95%CI)	p	OR (95%CI)	p	OR (95%CI)	p
Sweet Index_weighted	1.10 (0.99, 1.23)	0.09	1.18 (1.03, 1.37)	0.02	1.19 (1.06, 1.35)	0.005	1.26 (1.09, 1.45)	0.002
Time	1.09 (0.92, 1.28)	0.33	1.23 (1.01, 1.50)	0.04	1.14 (0.98, 1.33)	0.04	1.19 (0.99, 1.42)	0.06
Sweet Index x Time	1.00 (0.99, 1.00)	0.14	**0.99 (0.98, 1.00)**	**0.02**	**0.99 (0.98, 1.00)**	**0.002**	**0.99 (0.98, 0.99)**	**0.001**
Non-sweet Index_weighted	1.00 (0.96, 1.03)	0.84	1.04 (1.00, 1.09)	0.04	1.00 (0.97, 1.04)	0.87	1.01 (0.98, 1.05)	0.59
Time	0.98 (0.81, 1.19)	0.85	1.20 (0.97, 1.49)	0.09	0.95 (0.80, 1.13)	0.58	0.98 (0.80, 1.19)	0.81
Non-sweet index x Time	1.00 (1.00, 1.00)	0.67	1.00 (1.00, 1.00)	0.15	1.00 (1.00, 1.00)	0.81	1.00 (1.00, 1.00)	0.99

Supplementary Table S3. Cross-sectional analysis of the associations between snack indices and oral microorganism carriage

	S. mutans carriage__Saliva		*S. mutans* carriage__Plaque		*Candida* carriage__Saliva		*Candida* carriage__Plaque	
	OR (95%CI)	p	OR (95%CI)	p	OR (95%CI)	p	OR (95%CI)	p
Sweet Index								
12 months	1.13 (0.78, 1.64)	0.51	1.29 (0.89, 1.88)	0.18	1.21 (0.92, 1.60)	0.17	1.31 (0.96, 1.80)	0.09
18 months	1.21 (0.88, 1.67)	0.24	1.24 (0.87, 1.75)	0.23	0.92 (0.68, 1.24)	0.57	1.07 (0.72, 1.58)	0.74
24 months	0.92 (0.70, 1.21)	0.55	0.91 (0.70, 1.19)	0.49	**0.73 (0.55, 0.98)**	**0.04**	0.72 (0.51, 1.00)	0.05
Non-sweet Index								
12 months	0.73 (0.47, 1.12)	0.15	1.00 (0.64, 1.56)	0.99	1.13 (0.83, 1.52)	0.44	0.92 (0.66, 1.28)	0.61
18 months	1.61 (0.91, 2.87)	0.11	**3.18 (1.44, 7.01)**	**0.004**	1.07 (0.63, 1.83)	0.80	0.54 (0.26, 1.13)	0.10
24 months	1.11 (0.71, 1.73)	0.64	1.39 (0.90, 2.15)	0.13	1.05 (0.69, 1.60)	0.83	0.79 (0.49, 1.29)	0.35

Supplementary Figures

Supplementary Figure S1. Rank of the predictive factors of *S. mutans* carriage in saliva by age.

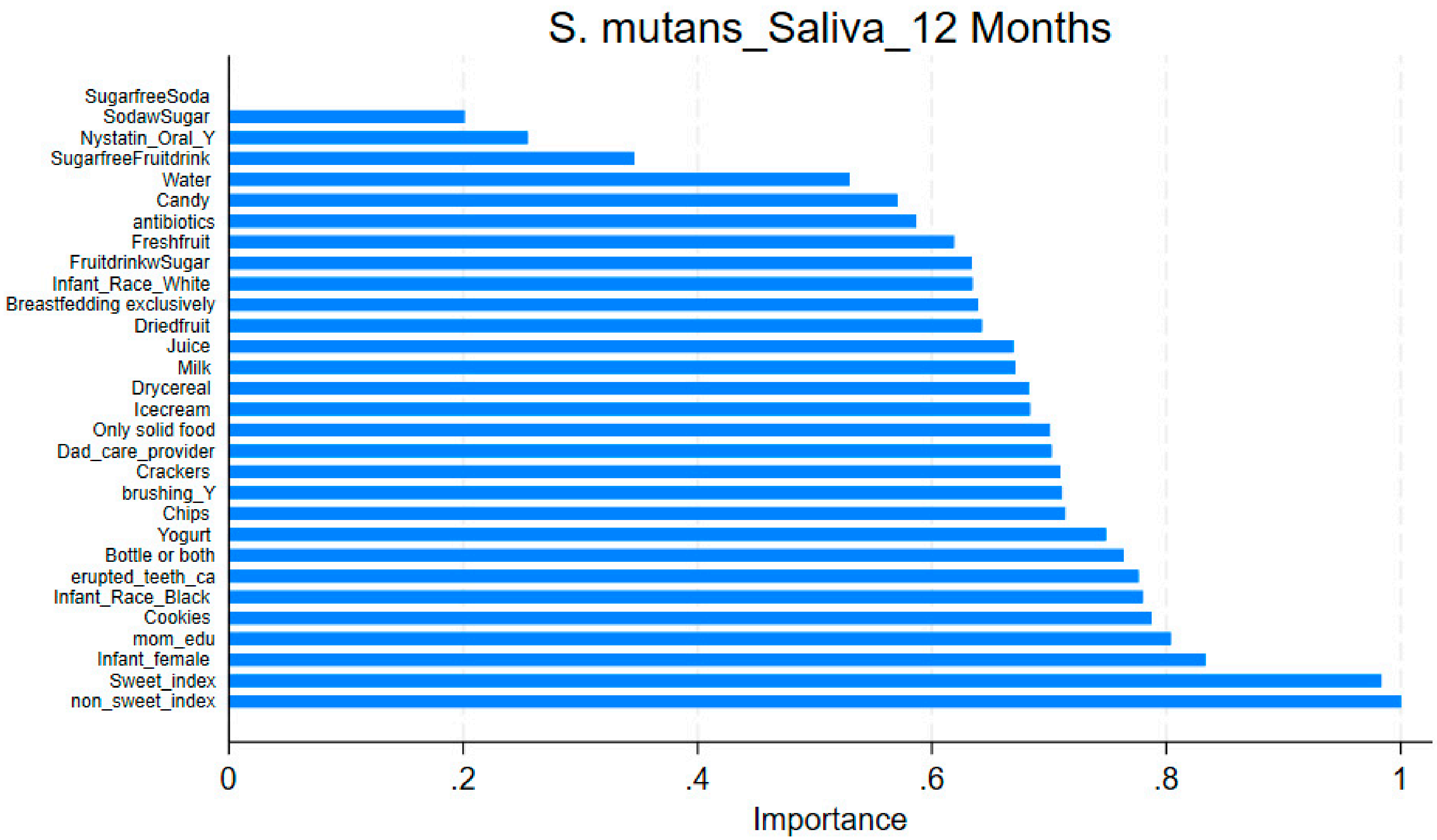

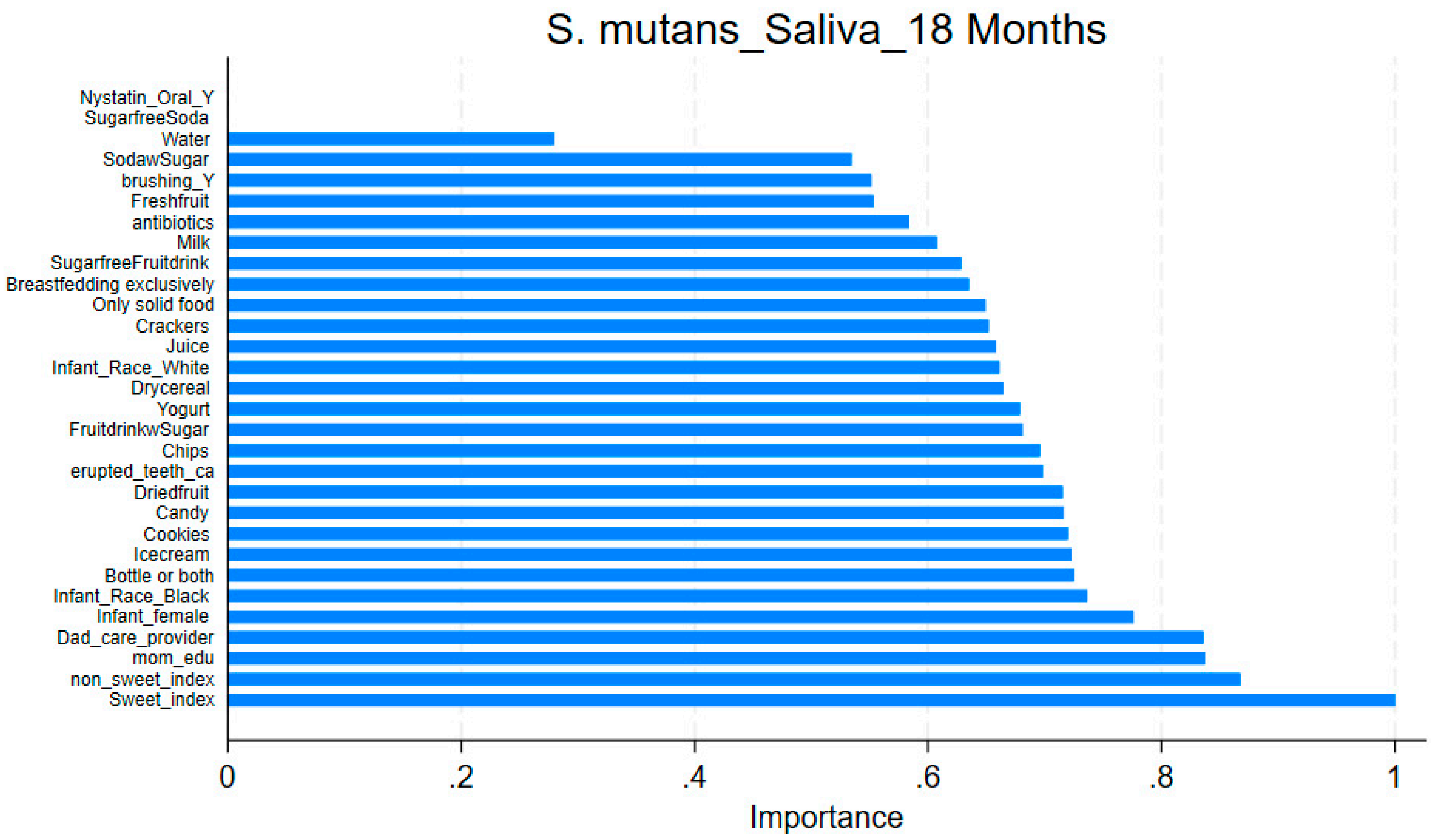
S. mutans_Saliva_18 Months
Nystatin_Oral_Y
SugarfreeSoda
Water
SodawSugar
brushing_Y
Freshfruit
antibiotics
Milk
SugarfreeFruitdrink
Breastfedding exclusively
Only solid food
Crackers
Juice
Infant_Race_White
Drycereal
Yogurt
FruitdrinkwSugar
Chips
erupted_teeth_ca
Driedfruit
Candy
Cookies
Icecream
Bottle or both
Infant_Race_Black
Infant_female
Dad_care_provider
mom_edu
non_sweet_index
Sweet_index
0
.2
.4
.6
.8
1
Importance

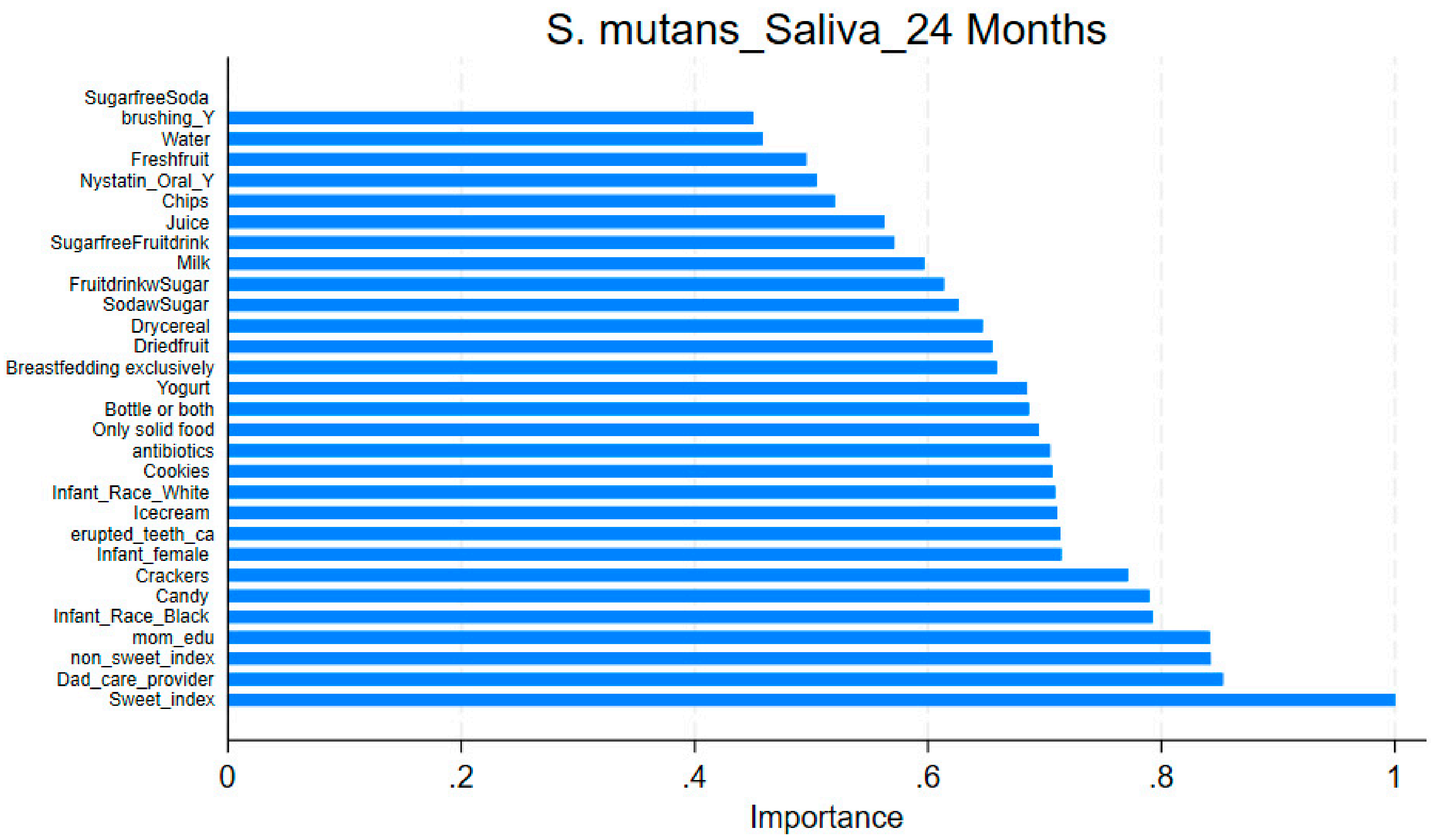
S. mutans_Saliva_24 Months
SugarfreeSoda
brushing_Y
Water
Freshfruit
Nystatin_Oral_Y
Chips
Juice
SugarfreeFruitdrink
Milk
FruitdrinkwSugar
SodawSugar
Drycereal
Driedfruit
Breastfedding exclusively
Yogurt
Bottle or both
Only solid food
antibiotics
Cookies
Infant_Race_White
Icecream
erupted_teeth_ca
Infant_female
Crackers
Candy
Infant_Race_Black
mom_edu
non_sweet_index
Dad_care_provider
Sweet_index
0
.2
.4
.6
.8
1
Importance

Supplementary Figure S2. Rank of the predictive factors of *S. mutans* carriage in plaque by age.

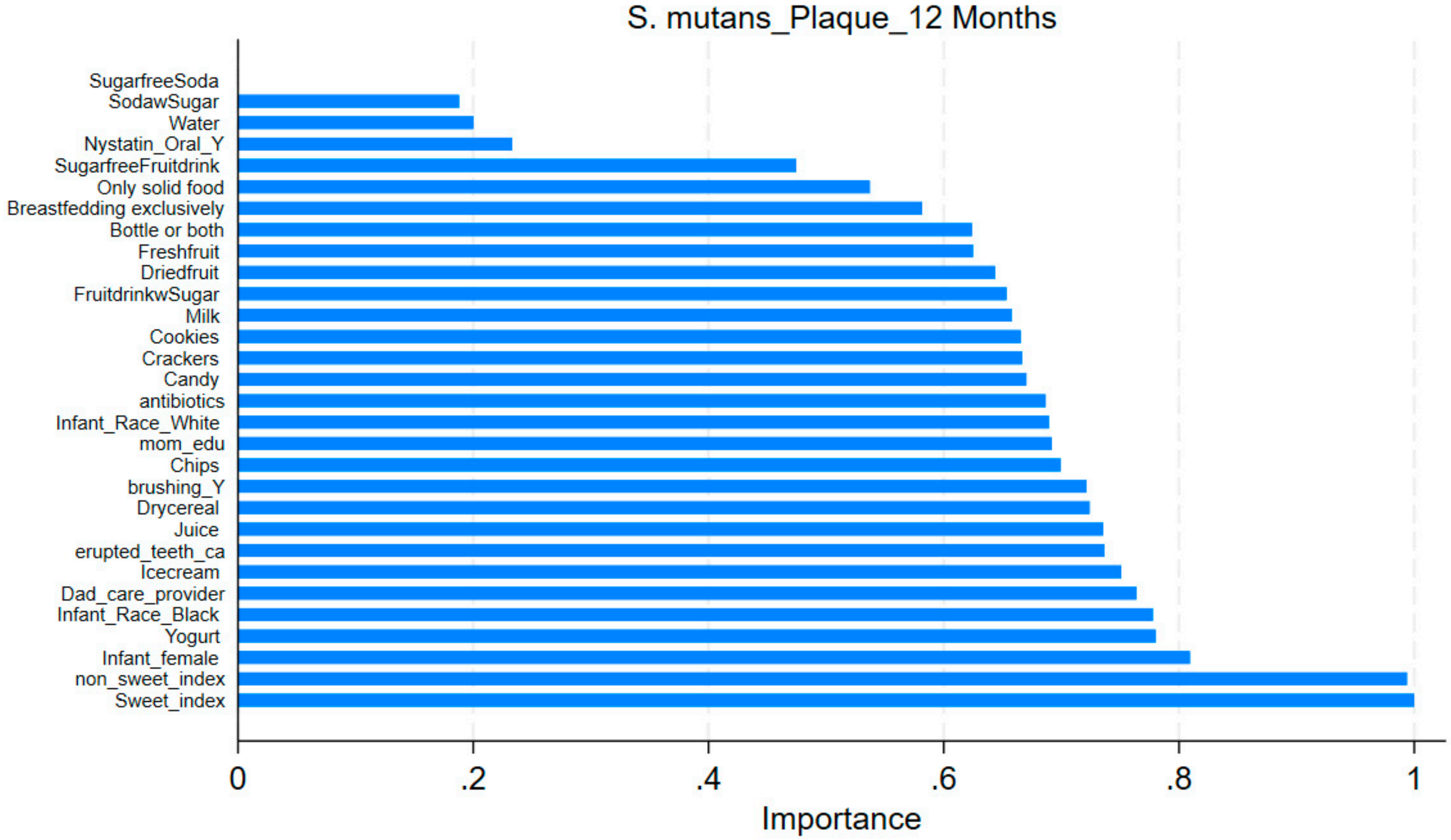

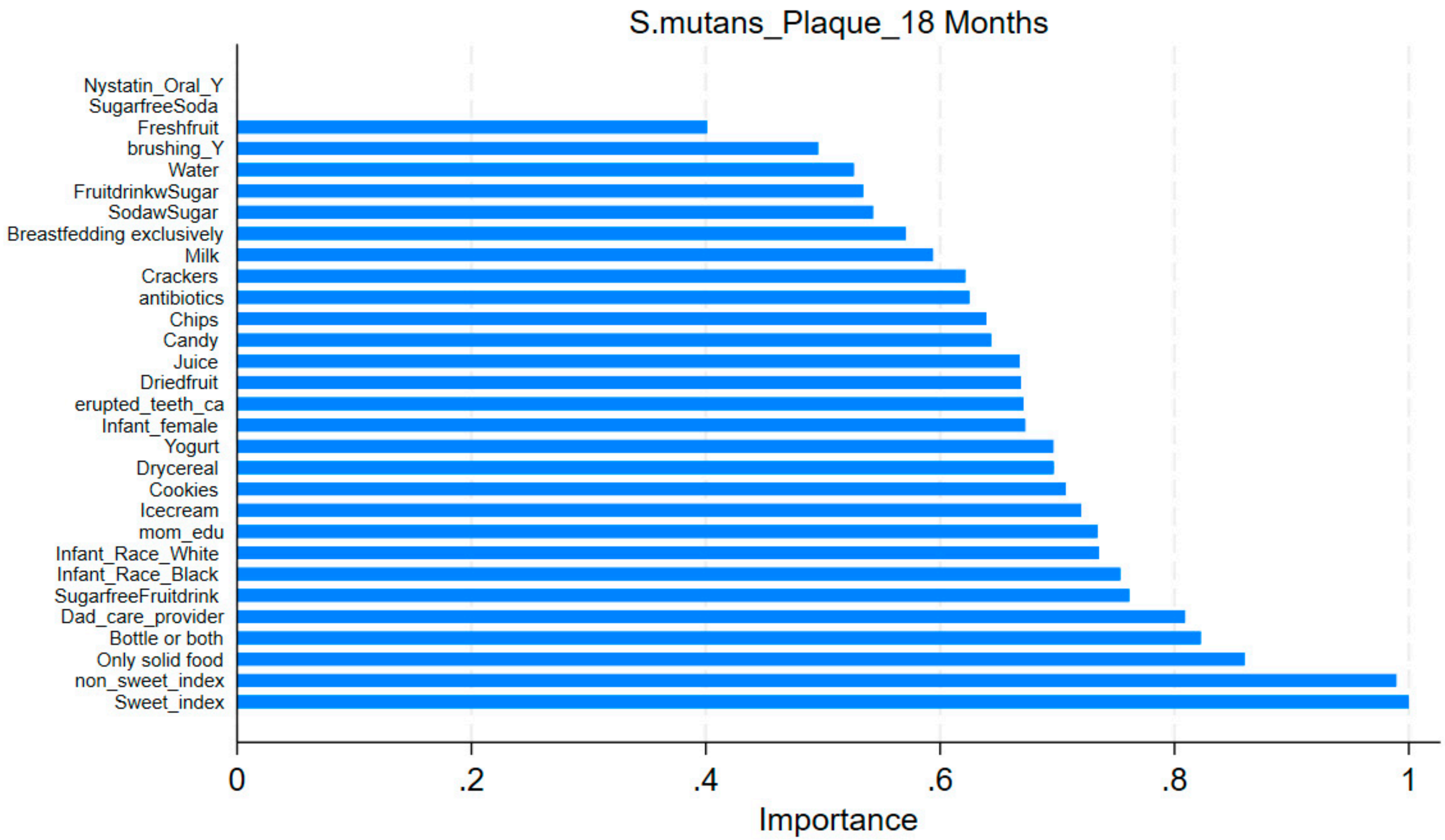
S.mutans_Plaque_18 Months
Nystatin_Oral_Y
SugarfreeSoda
Freshfruit
brushing_Y
Water
FruitdrinkwSugar
SodawSugar
Breastfedding exclusively
Milk
Crackers
antibiotics
Chips
Candy
Juice
Driedfruit
erupted_teeth_ca
Infant_female
Yogurt
Drycereal
Cookies
Icecream
mom_edu
Infant_Race_White
Infant_Race_Black
SugarfreeFruitdrink
Dad_care_provider
Bottle or both
Only solid food
non_sweet_index
Sweet_index
0
.2
.4
.6
.8
1
Importance

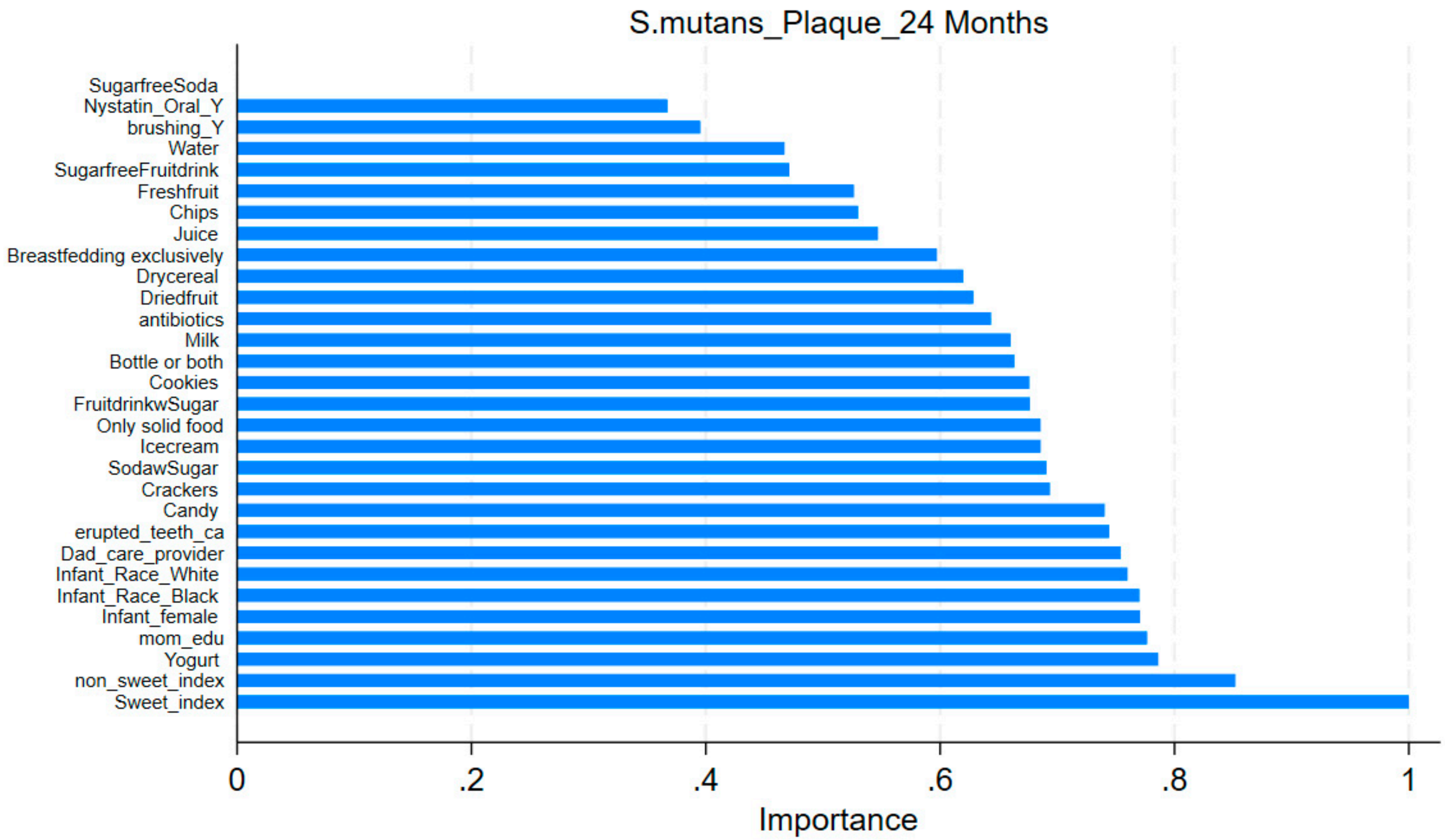
S.mutans_Plaque_24 Months
SugarfreeSoda
Nystatin_Oral_Y
brushing_Y
Water
SugarfreeFruitdrink
Freshfruit
Chips
Juice
Breastfedding exclusively
Drycereal
Driedfruit
antibiotics
Milk
Bottle or both
Cookies
FruitdrinkwSugar
Only solid food
Icecream
SodawSugar
Crackers
Candy
erupted_teeth_ca
Dad_care_provider
Infant_Race_White
Infant_Race_Black
Infant_female
mom_edu
Yogurt
non_sweet_index
Sweet_index
0
.2
.4
.6
.8
1
Importance

Supplementary Figure S3. Rank of the predictive factors of *Candida* carriage in saliva by age.

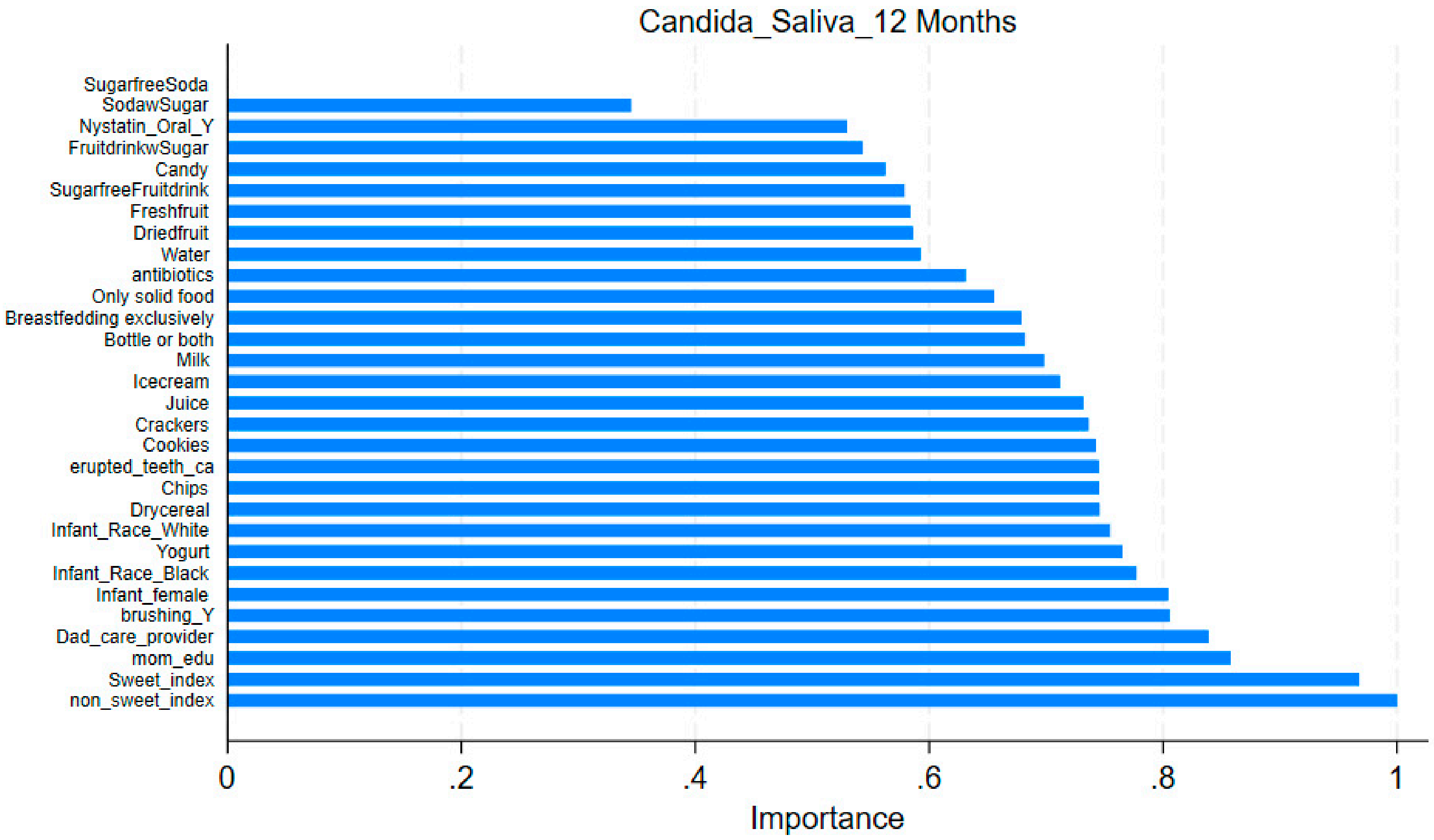

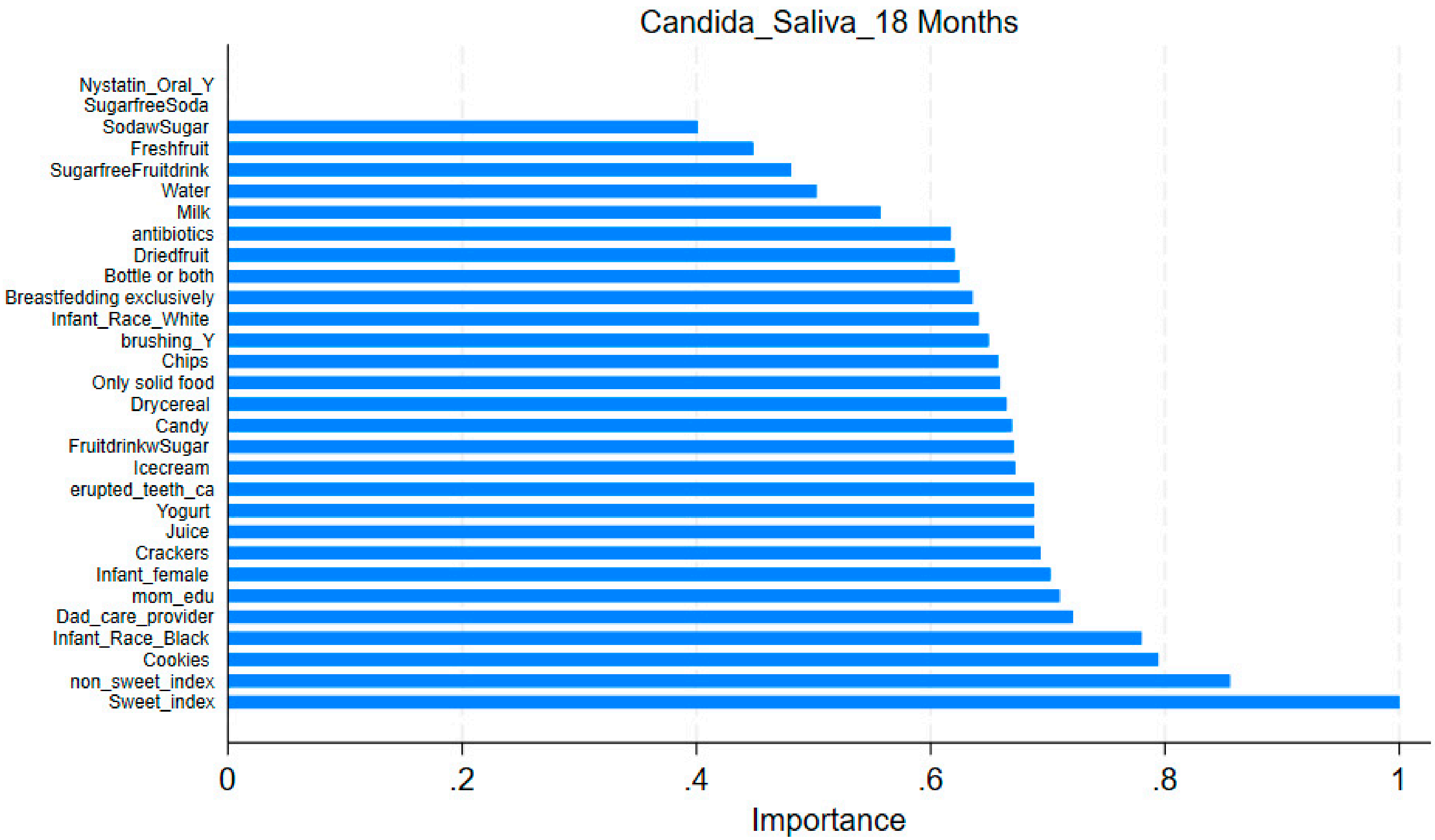
Candida_Saliva_18 Months
Nystatin_Oral_Y
SugarfreeSoda
SodawSugar
Freshfruit
SugarfreeFruitdrink
Water
Milk
antibiotics
Driedfruit
Bottle or both
Breastfedding exclusively
Infant_Race_White
brushing_Y
Chips
Only solid food
Drycereal
Candy
FruitdrinkwSugar
Icecream
erupted_teeth_ca
Yogurt
Juice
Crackers
Infant_female
mom_edu
Dad_care_provider
Infant_Race_Black
Cookies
non_sweet_index
Sweet_index
0
.2
.4
.6
.8
1
Importance

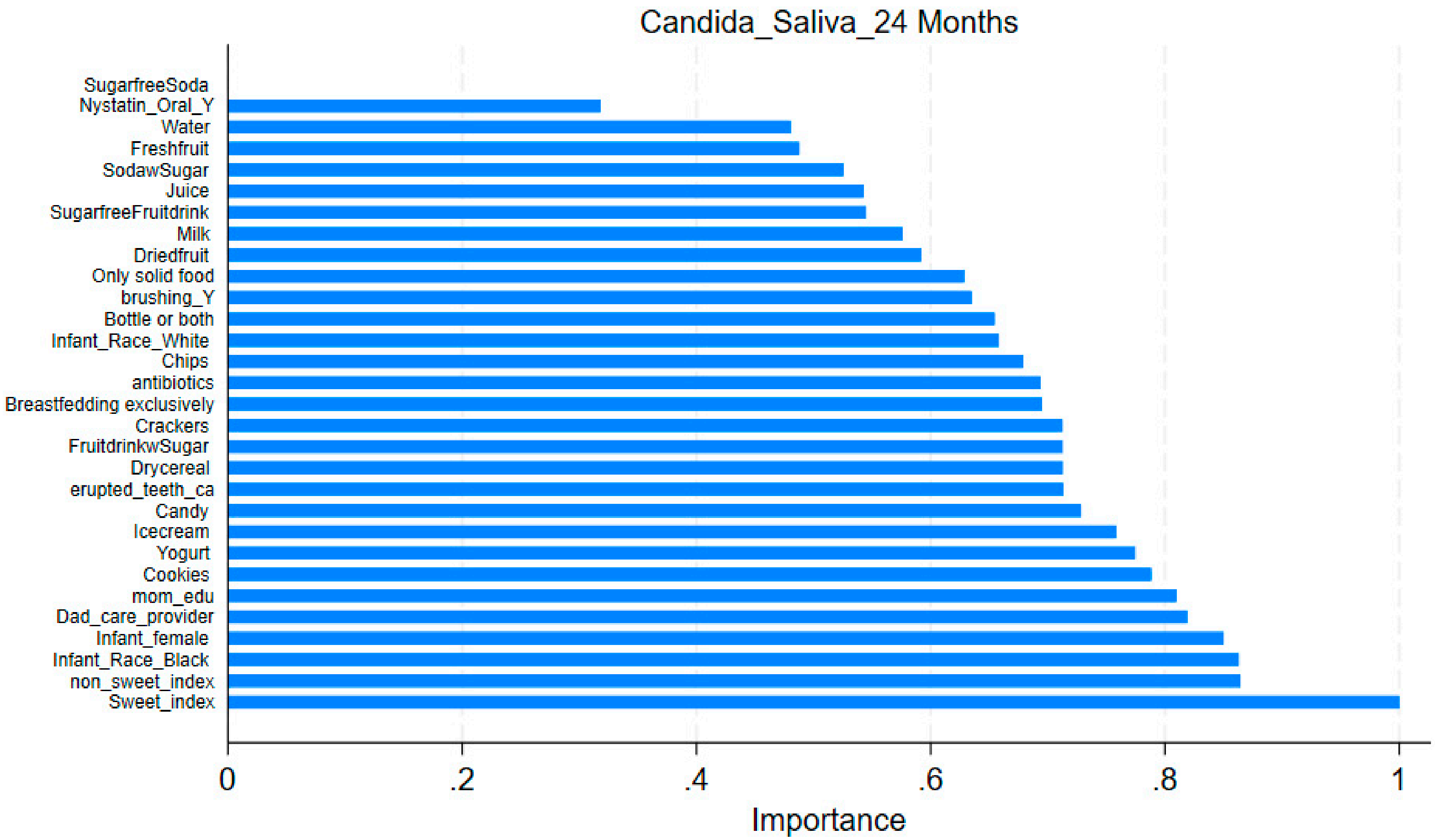
Candida_Saliva_24 Months
SugarfreeSoda
Nystatin_Oral_Y
Water
Freshfruit
SodawSugar
Juice
SugarfreeFruitdrink
Milk
Driedfruit
Only solid food
brushing_Y
Bottle or both
Infant_Race_White
Chips
antibiotics
Breastfedding exclusively
Crackers
FruitdrinkwSugar
Drycereal
erupted_teeth_ca
Candy
Icecream
Yogurt
Cookies
mom_edu
Dad_care_provider
Infant_female
Infant_Race_Black
non_sweet_index
Sweet_index
0
.2
.4
.6
.8
1
Importance

Supplementary Figure S4. Rank of the predictive factors of *Candida* carriage in plaque by age.

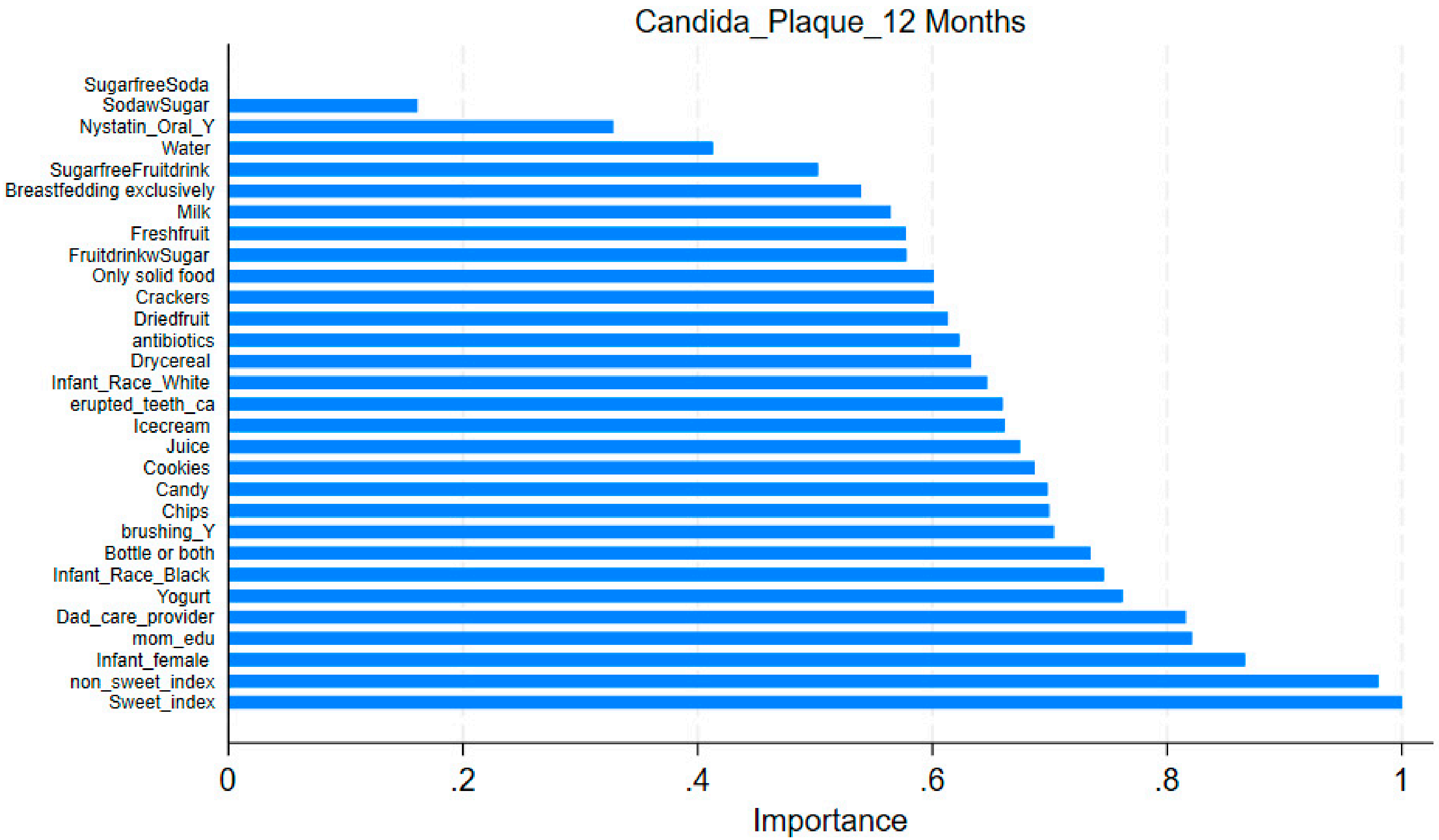

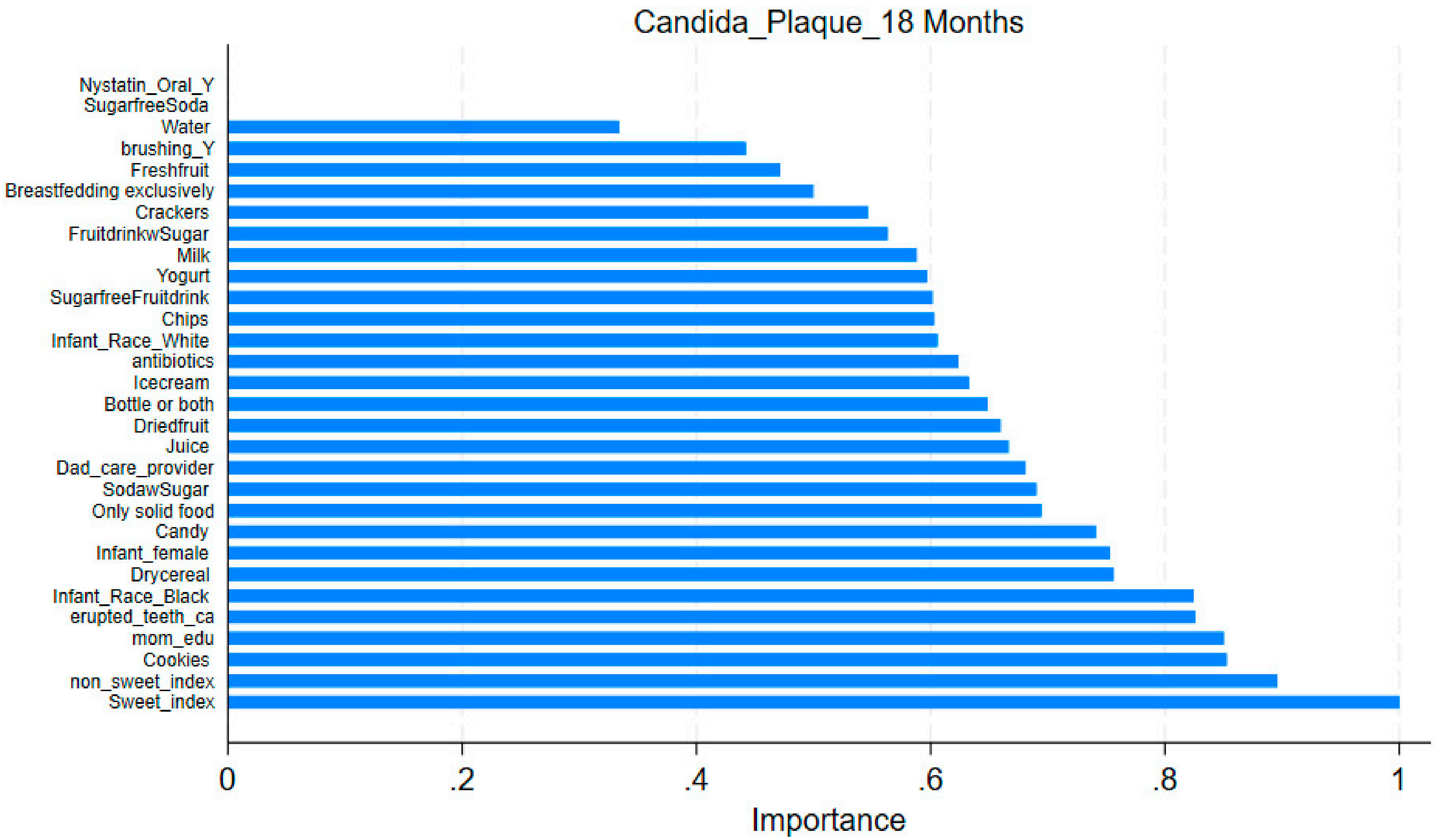

Candida_Plaque_18 Months
Nystatin_Oral_Y
SugarfreeSoda
Water
brushing_Y
Freshfruit
Breastfedding exclusively
Crackers
FruitdrinkwSugar
Milk
Yogurt
SugarfreeFruitdrink
Chips
Infant_Race_White
antibiotics
Icecream
Bottle or both
Driedfruit
Juice
Dad_care_provider
SodawSugar
Only solid food
Candy
Infant_female
Drycereal
Infant_Race_Black
erupted_teeth_ca
mom_edu
Cookies
non_sweet_index
Sweet_index
0
.2
.4
.6
.8
1
Importance

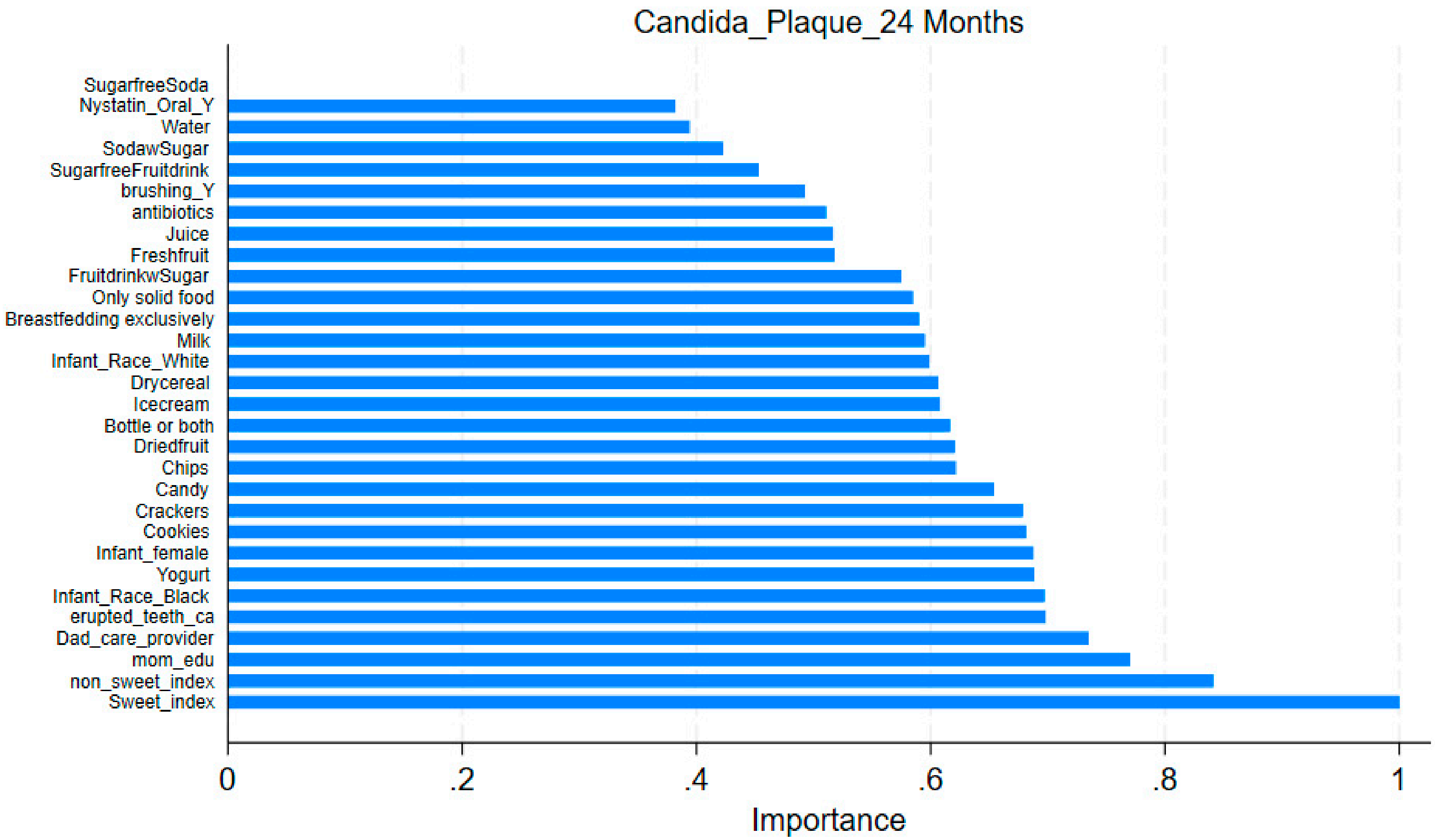
Candida_Plaque_24 Months
SugarfreeSoda
Nystatin_Oral_Y
Water
SodawSugar
SugarfreeFruitdrink
brushing_Y
antibiotics
Juice
Freshfruit
FruitdrinkwSugar
Only solid food
Breastfedding exclusively
Milk
Infant_Race_White
Drycereal
Icecream
Bottle or both
Driedfruit
Chips
Candy
Crackers
Cookies
Infant_female
Yogurt
Infant_Race_Black
erupted_teeth_ca
Dad_care_provider
mom_edu
non_sweet_index
Sweet_index
0
.2
.4
.6
.8
1
Importance
